# Municipality and Adjusted Gross Income Influence Outcome of Patients Diagnosed with Pancreatic Cancer in a Newly Developed Cancer Center in Mercer County New Jersey, USA, a Single Center Study

**DOI:** 10.3390/cancers13071498

**Published:** 2021-03-24

**Authors:** Cataldo Doria, Patrick De Deyne, Sherry Dolan, Jooyeun Chung, Karen Yatcilla, Ladan Zarifian, Rona Remstein, Eric Schwartz

**Affiliations:** Capital Health, Medical Director Cancer Center, 1 Capital Way, Pennington, NJ 08534, USA; PDeDeyne@capitalhealth.org (P.D.D.); SDolan@capitalhealth.org (S.D.); JChung2@capitalhealth.org (J.C.); KYatcilla@capitalhealth.org (K.Y.); LZarifian@capitalhealth.org (L.Z.); RHRemstein@capitalhealth.org (R.R.); ESchwartz2@capitalhealth.org (E.S.)

**Keywords:** pancreatic cancer, disparity, outcome

## Abstract

**Simple Summary:**

In the study we used a patient registry and associated its data with geography and tax returns to assess the impact of socioeconomic status and race on clinical outcomes in patients with pancreatic cancer. Our findings indicate that African Americans and patients who live in an area with low socioeconomic status have significantly lower overall survival and lower utilization of specialty services. Our premise is that these patients encounter barriers when accessing specialty services at larger academic medical centers and that regional medical centers have a critical role to play in providing specialty services to the community.

**Abstract:**

Socioeconomic status (SES) correlates directly to ZIP code. Mercer County is not atypical as a collection of a dozen municipalities with a suburban/metropolitan population of 370,430 in the immediate vicinity of a major medical center. The purpose of this study for Mercer County, New Jersey, USA is to determine whether a patient’s ZIP code is related to the outlook of pancreatic cancer defined as staging at diagnosis, prevalence, overall survival, type of insurance, and recurrence. Our hypothesis was that specific variables such as socio-economic status or race could be linked to the outcome of patients with pancreatic cancer. We interrogated a convenience sample from our cancer center registry and obtained 479 subjects diagnosed with pancreatic cancer in 1998-2018. We selected 339 subjects by ZIP code, representing the plurality of the cases in our catchment area. The outcome variable was overall survival; predictor variables were socio-economic status (SES), recurrence, insurance, type of treatment, gender, cancer stage, age, and race. We converted ZIP code to municipality and culled data using adjusted gross income (AGI, FY 2017). Comparative statistical analysis was performed using chi-square tests for nominal and ordinal variables, and a two-way ANOVA test was used for continuous variables; the p-value was set at 0.05. Our analysis confirmed that overall survival was significantly higher for Whites and for individuals who live in a municipality with a high SES. Tumor stage at the time of diagnosis was not different among race and SES; however, statistically significant differences for race or SES existed in the type of treatment received, with disparities found in those who received radiation therapy and surgery but not chemotherapy. The data may point to a lack of access to specific care modalities that subsequently may lead to lower survival in an underserved population. Access to care, optimal nutritional status, overall fitness, and co-morbidities could play a major role and confound the results. Our study suggests that low SES has a negative impact on overall pancreatic cancer survival. Surgery for pancreatic cancer should be appropriately decentralized to those community cancer centers that possess the expertise and the infrastructure to carry out specialized treatments regardless of race, ethnicity, SES, and insurance.

## 1. Introduction

Globally, pancreatic cancer accounts for 4% of all cancer-related deaths. In the USA, it accounts for 2.7% of all new cancer cases. However, it is projected that the cancer burden for pancreatic cancer will become the second largest cause of cancer-related deaths by 2030 [1]. Approximately 47,000 people die of pancreatic cancer every year in the US [2]. Five-year relative survival after diagnosis for patients with pancreatic cancer is still dismal (10%) and unchanged over the last several decades. Disparities in pancreatic cancer outcomes have been studied previously in the USA and point to modifiable and non-modifiable factors such as tobacco smoking, diabetes, and obesity, risk factors that in the US are more commonly seen in African Americans [3]. Genetic predisposition has also been proven to play a role in the genesis of pancreatic cancer [4]. Currently, approximately 30% of patients with pancreatic adenocarcinoma receive surgical treatment, which is the single most effective treatment for pancreatic cancer [5]. Surgical resection can be associated with neo-adjuvant chemo or chemo-radiation [6,7]; it is always administered with adjuvant chemotherapy, and sometimes adjuvant chemo-radiation treatment [8]. The indications and contra-indications to surgery have changed over the years, where patients that were previously considered non-resectable, because of the involvement of major vascular structures, have now become resectable following neo-adjuvant chemo-radiation [9,10]. This approach has led us to offer surgical treatment to an increased number of patients [11]. The presence of disparities in cancer treatment is well recognized and documented [12,13]. When looking specifically at pancreatic cancer, numerous studies have documented that in the United States (US), African Americans (AAs) remain at a disadvantage compared to other races and ethnicities in terms of outcomes when diagnosed with pancreatic cancer [14,15]. With respect to applying widely reported global and national data, there may be local intricacies present that require a more granular approach that may apply to a community cancer center; this nuance in outcomes based on local geographies was shown previously [15,16]. We believe that our approach in further studying this issue is pertinent because municipality is a unique source of learning, and, together with adjusted gross income (AGI), has never been used before to confirm an underlying truth. Additionally, we think that a single center study in a newly formed community cancer center gives additional insight into the issue of disparity and discrimination of cancer patients, and allows us to design community specific interventions.

## 2. Materials and Methods

Study Population: Data from hospital-based registries and other sources are submitted to population-based registries including, in the US, state cancer registries, and the National Cancer Database. The uniform data set used is determined by the North American Association of Central Cancer Registries (NAACCR) and we followed their definitions for each variable. To define stage, we used the National Cancer Institute Surveillance Epidemiology and End Results Reporting Program (NCI SEER), grouped as: in situ, local, regional (direct, regional lymph node, or both), or distant. We used our internal cancer registry, which captures cancer diagnosis, treatment, and follow-up for every cancer case in a population. In an initial evaluation, we collected data from patients diagnosed with pancreatic cancer from 1998 until 2018 residing in Mercer County, New Jersey, with 12 municipalities, and a suburban/urban population of 367,430 as of 2019. This approach captured 339 of 479 cases (71%) and allowed us to generate a convenience sample and initiate a retrospective, single site, cross-sectional study to formulate our hypothesis. This was the first analysis of our internal database and we did not opt for a cohort design, since we did not establish the predictor variables prior to the start of the study and, moreover, the data were extracted in an aggregate format. To properly address subjects by race, we used the US Census designation for race, where “White” indicates a person having origins in any of the original peoples of Europe, the Middle East, or North Africa. It includes people who indicate their race as “White” or report entries such as Irish, German, Italian, Lebanese, Arab, Moroccan, or Caucasian, and “Black or African American” refers to a person having origins in any of the Black racial groups of Africa. It includes people who indicate their race as “Black or African American,” or report entries such as African American, Kenyan, Nigerian, or Haitian [17,18].

Evaluation of Socioeconomic Status (SES): To determine which ZIP codes were classified as high, Middle, and low SES in Mercer County, NJ, we culled data from individual income tax returns: selected income and tax items by state, ZIP code, and size of adjusted gross income (AGI), tax year 2017, which included the number of tax returns (cases) by ZIP code by adjusted gross income reported on all 2017 federal income tax returns (Table 1). While we collected the clinical and hospital data of subjects treated between 1998 and 2018, we limited ourselves to retrieving income data to the tax year 2018, thereby assuming that few or minimal changes in SES would have occurred between 1998 and 2017 in these municipalities. The data are obtained in 6 categories: USD 1 to under USD 25,000, USD 25,000 to under USD 50,000, USD 50,000 to under USD 75,000, USD 75,000 to under USD 100,000, USD 100,000 to under USD 200,000, and USD 200,000 or more. We selected ZIP codes from subjects who resided in one of the 21 ZIP codes for Mercer County, NJ and recoded them into specific groups of SES. Based on a median AGI of USD 54,000 in Mercer County, NJ, we created groups using a cutoff at filings of <USD 25,000 or >USD 75,000, leading to three groups: low SES = municipalities where the majority of the filings were less than USD 25,000, mid SES = municipalities where the majority of the filings were between USD 25,000 and USD 75,000, high SES = municipalities where the majority of returns were over USD 75,000. 

## 3. Statistical Analysis

We were interested in identifying differences in outcome such as overall survival based on the type of treatment received, type of insurance, and tumor stage at the time of diagnosis and analyzed whether we could find differences in function of the SES or race. We obtained aggregated data from our internal cancer registry and selected patients who were diagnosed with pancreatic cancer. The database was cleaned and if data points were not obtained, they were recoded as missing in the statistical software (SPSS, v26) that was used to create frequency tables for the nominal and ordinal variables and descriptive statistics to calculate the means and variance (including standard deviation, standard error) for continuous variables. Statistical relationships and comparisons between groups were performed using chi-Square tests for nominal and ordinal variables, and a two-way ANOVA test was used for continuous variables; for each series of analyses, the *p*-value was set at 0.05 to determine significant differences. Inferential statistics to assess relationships between variables that would be predictive of overall survival (OS) were not performed. 

## 4. Results

The overall and general characteristics of the sample are summarized in Table 2. We captured race, Hispanic ethnicity, gender, insurance, stage at diagnosis, SES, the type of medical or surgical service that the patients received inside or outside our hospital system (diagnosis, chemotherapy, surgery, radiation therapy, palliative care), status (alive according to latest visit or confirmed dead), the type of recurrence, age at the time of diagnosis, and overall survival. Follow-up time for the whole cohort was 368 ± 608 days (mean ± standard deviation). Our data indicate similarities but also differences when compared to the national profile obtained from comprehensive national SEER data [17] and, for instance, our sample contained 38% AAs, which may be more typical for a regional cancer center caring for a combined urban and suburban population. Results (Table 3) clearly indicated significant differences in overall survival (OS) in groups identified as living in areas of low SES and being non-White, which is not surprising and has been shown previously [17]. Our data show that OS was reduced from 16.6 months in the high SES group to 9.2 months in the mid SES group and 8.7 months in the low SES group. Similarly, OS for Asians was 5.8 months, and for African Americans was 7.5 months compared to Whites, with an OS of 13.4 months. To further study any associations between SES or race with other variables, we systematically analyzed the nominal variables statistically. We focused on type of treatment, insurance coverage, and American Joint Commission on Cancer (AJCC) stage at diagnosis. When we asked whether the type of treatment received was dependent on SES or race, we obtained a mixed picture. There was no statistical difference in the distribution of the number of subjects from a different SES (low, mid, high) when they were diagnosed, received chemotherapy, surgery, or were referred to palliative care (Table 4). However, we observed a non-random distribution between radiation therapy and SES, with fewer patients receiving radiation therapy in the low SES group (24%) and mid SES group (22%) compared to 37% in the high SES group. Similarly, when we asked whether the distribution of the type of treatment received was different by race, we noted a unique pattern. Significant differences were not detected between race and being diagnosed, receiving chemotherapy, radiation therapy, or receiving palliative care (Table 4). However, we noted a significant non-random distribution between the numbers of subjects who received surgery and race, with 20% of Asians opting for surgery, while only 14% of African Americans received surgery compared to double that number (29%) in Whites. Similarly, the type of insurance showed significant differences based on the SES and race (Table 5A,B). Interestingly, SES and race were not statistically and differentially distributed when assessed against the AJCC stage at the time of diagnosis (Table 6A,B). The latter is an important point and sheds light on the fact the OS seems to be affected especially by SES (as a surrogate for income) or race but not by staging at diagnosis. Combined with the differences in type of insurance, race, or SES (Table 5A,B), these data in the study point to healthcare inequities that may be associated with access to healthcare, level of healthcare insurance and coverage, bias towards receiving or being informed about selected treatments (surgery), and perhaps other reasons. It is possible that these factors are major contributors in the lower OS that we noted in individuals who live in a municipality with low SES or are non-White in the USA. 

## 5. Discussion

Previous studies addressing disparities in pancreatic cancer treatment and outcome have used state [19,20], national [21], or international [22,23], databases such as SEER, and others [13,16], to address their respective hypotheses. Databases share common flaws such as incorrect entries, missed entries, etc. that complicate the analysis of large data sets. Although our study is also based on the analysis of data extracted from a database, we think that a single center cancer registry offers a statistically reduced chance of error. Furthermore, we believe that our newly developed community cancer center carries a number of unique features that makes our case stronger. Our metropolitan campus is more than 100 years old, and is located in the City of Trenton, New Jersey. The Regional Medical Center (RMC) has historically provided care to the underserved and has gained respect and experience in the community [18]. In addition, our approach of looking at municipalities and adjusted gross income to study how SES contributes to the disparity in treatment and outcomes of pancreatic cancer is one that has been addressed [12,18] but not clearly demonstrated [18]. Results of these studies are somewhat controversial [12].

Previous studies done in the US have invariably shown that AAs have a higher prevalence of the most commonly known risk factors for developing pancreatic cancer, namely, obesity, diabetes, and tobacco usage [13,24]. They are, therefore, by definition, at higher risk of developing pancreatic cancer when compared to other races and ethnicities [5,13,24]. A common trend has also been shown of AAs not being treated as often as they should with surgical resection, the single most effective treatment for pancreatic cancer [5]. This trend has been attributed to multiple possible causes, such as access to care [5,19], stage at diagnosis [20], interpreting the standard of care [21], nutritional status [22], and overall fitness [23]. It is our opinion that at least a component of the existing disparity in the outcome of pancreatic cancer has been, and is currently, fueled by the assumption that pancreatic surgery should only be done in major academic medical centers because the expertise and the infrastructures needed to treat these patients only exist in those settings. The problem is that uninsured and underinsured patients constitute a significant portion of patients in the low SES group, and therefore may not have access to the single most effective treatment for pancreatic cancer, namely, surgical resection. These patients, in fact, may not have the means to travel to the major academic medical centers and if they are able to find transportation, their care may be denied due to lack of coverage [18]. We believe that the answer to counteract the proven disparity in outcome of patients diagnosed with pancreatic cancer is in fact the opposite of what has been historically recommended [25,26]. Patients should stay local and should be treated in community cancer centers with proven expertise in the field and proper infrastructures. This approach will result in a generalized cost-containing effort. 

## 6. Conclusions

Numerous studies have suggested that SES and race are powerful determinants affecting the type of treatment a patient with resectable pancreatic cancer may receive as well as the impact on their ultimate outcome and survival [12,27]. African Americans consistently have lower rates of surgical resection, despite controlling for stage of disease [5,28,29]. Location and distance to travel are integral during patient decision making and should be considered when making the assumption that surgical resections performed only at tertiary care centers are associated with better outcomes [30]. Distance decay association demonstrates that patients living further away from healthcare facilities have worse health outcomes than those who live closer [31]. A study in a VA population showed that if operative mortality risk at a local hospital were the same as the regional hospital, all patients surveyed preferred local surgery and if local mortality were twice the risk of the regional center, 45% would still prefer the local surgery [32]. The impact of social determinants of health, for which transportation or distance traveled could serve as a proxy, can be linked with hospital quality measures to better care for patients. The notion that academic medical centers are the only destination for surgical treatment for pancreatic cancer may ignore the existence of barriers to care created by SES and geography. Evidence shows that safety-net hospitals, with a focus on vulnerable populations, are able to provide care with outcomes similar to the national average [18]. 

A limitation in our study is not accounting for the progressive technical improvements that may have affected outcomes between 1998 and 2018. For instance, the use of single beam radiation therapy became more prevalent and, similarly, new types of chemotherapy were introduced during that time, which may have created a confounding effect in our study. Moreover, our sample included subjects who may have received only part of their care locally and it is conceivable that subjects who were referred to an academic medical center may have chosen not to undergo surgery because of the threshold to travel, hence influencing the results. 

In conclusion, by using location (ZIP code) and income as a surrogate for SES, we were able to confirm that patients with a low SES have worse OS when diagnosed with pancreatic cancer. We believe that the practice of centralizing pancreatic surgery in major academic medical centers may be partially responsible for the disparity in outcomes of patients with different SESs. In addition, the cost of care is much more expensive in major academic medical centers, causing an increased burden on society as a whole [33,34,35]. Our recommendation is to appropriately decentralize pancreatic surgery to those community cancer centers that possess the expertise and the infrastructure to carry out specialized treatments regardless of the race, ethnicity, SES, and insurance, and will do so at a fraction of the cost. Progressive organizations should be federally supported to pursue the need to further study the role of combined surgical outcomes and travel distance for care as a mechanism to overcome established disparities that exist in pancreatic cancer treatment outcomes. 

## Figures and Tables

**Table 1:** Socioeconomic status (SES) strata created from ZIP code and adjusted gross income (AGI). Municipalities with their ZIP code, including proportion of tax returns (AGI from 2017) and their adjudication according to socioeconomic status in low (<USD 25,000), mid (USD 25,000–USD 75,000), or high (>USD 75,000) income brackets. The overall distribution of the subjects across the study was as follows: low SES 35.7% (n = 121 subjects), mid SES 37.2% (n = 126 subjects), and high SES 27.1% (n = 92 subjects). We recoded the cancer database into SES strata and analyzed several clinically relevant variables.

ZIP Code, Municipality, Number of Pancreatic Cancer Subjects in Sample (Total N = 328)	SES	Adjusted Gross Income	% of Tax Returns	Total Tax Returns
08525 Hopewell (n = 2)	High	USD 1 under USD 25,000	22.32%	2330
		USD 25,000 under USD 75,000	24.04%	
		USD 75,000 and above	53.65%	
08534 Pennington (n = 10)	High	USD 1 under USD 25,000	21.97	6280
		USD 25,000 under USD 75,000	20.07%	
		USD 75,000 and above	57.96%	
08540 Princeton (n = 5)	High	USD 1 under USD 25,000	21.06%	20,850
		USD 25,000 under USD 75,000	21.68%	
		USD 75,000 and above	57.27%	
08550 Princeton JCT (n = 7)	High	USD 1 under USD 25,000	20.46%	9190
		USD 25,000 under USD 75,000	16.97%	
		USD 75,000 and above	62.97%	
08520 Hightstown (n = 0)	High	USD 1 under USD 25,000	28.53%	14,370
		USD 25,000 under USD 75,000	35.01%	
		USD 75,000 and above	36.46%	
08560 Titusville (n = 0)	High	USD 1 under USD 25,000	22.03%	1770
		USD 25,000 under USD 75,000	22.59%	
		USD 75,000 and above	55.36%	
08608 Trenton (n = 1)	Low	USD 1 under USD 25,000	56.41%	390
		USD 25,000 under USD 75,000	43.59%	
		USD 75,000 and above	0%	
08609 Treton/hamilton (n = 12)	Low	USD 1 under USD 25,000	54.16%	5890
		USD 25,000 under USD 75,000	41.60%	
		USD 75,000 and above	4.24%	
08610 Trenton (n = 20)	Mid	USD 1 under USD 25,000	33.61%	15,950
		USD 25,000 under USD 75,000	44.95%	
		USD 75,000 and above	21.44%	
08611 Trenton (n = 28)	Mid	USD 1 under USD 25,000	54.60%	9890
		USD 25,000 under USD 75,000	40.14%	
		USD 75,000 and above	5.25%	
08618 Trenton/Hamilton/Ewing (n = 88)	Low	USD 1 under USD 25,000	43.55%	15,590
		USD 25,000 under USD 75,000	41.75%	
		USD 75,000 and above	14.69%	
08619 Mercerville (n = 20)	Mid	USD 1 under USD 25,000	26.47%	12,050
		USD 25,000 under USD 75,000	38.17%	
		USD 75,000 and above	35.35%	
08620 Hamilton/Yardville (n = 10)	High	USD 1 under USD 25,000	25.44%	6210
		USD 25,000 under USD 75,000	35.58%	
		USD 75,000 and above	38.97%	
08628 Ewing Hopewell (n = 18)	Mid	USD 1 under USD 25,000	21.52%	5250
		USD 25,000 under USD 75,000	40.00%	
		USD 75,000 and above	38.48%	
08629 Trenton/Hamilton (n = 6)	Mid	USD 1 under USD 25,000	43.51%	6160
		USD 25,000 under USD 75,000	47.73%	
		USD 75,000 and above	8.77%	
08638 Ewing (n = 56)	Mid	USD 1 under USD 25,000	39.80%	10,880
		USD 25,000 under USD 75,000	41.55%	
		USD 75,000 and above	18.66%	
08648 Lawrenceville (n = 32)	High	USD 1 under USD 25,000	26.16%	15,290
		USD 25,000 under USD 75,000	33.16%	
		USD 75,000 and above	40.68%	
08690 Hamilton SQ (n = 15)	High	USD 1 under USD 25,000	23.63%	10,620
		USD 25,000 under USD 75,000	33.52%	
		USD 75,000 and above	42.84%	
08691 Hamilton Robbinsville Windsor (n = 6)	High	USD 1 under USD 25,000	21.11%	7910
		USD 25,000 under USD 75,000	26.04%	
		USD 75,000 and above	52.85%	

**Table 2:** Demographics. Description of the total number subjects in the study, organized by: racial or ethnic characteristics, gender, type of insurance (payer), stage at the time of diagnosis, socioeconomic status, type of medical service or surgical service provided either internally or externally of the hospital (diagnosis, chemotherapy, surgery, radiation therapy, palliative care), status based on the most current information (based on the most recent visit), type of recurrence. Age and overall survival (OS) are shown as mean ± standard deviation.

Variable	Value	Count (% of Total)
**Race**	White African American Asian	204 (60.2) 130 (38.3) 5 (1.5)
**Hispanic**	Hispanic Non-Hispanic	14 (4.1) 325 (95.9)
**Gender**	Male Female	168 (49.6) 171 (50.4)
**Payer**	Insured, but not specified Medicare Medicaid Private Not insured	33 (9.7) 188 (55.5) 18 (5.3) 71 (20.9) 29 (8.6)
**Stage**	Stage 0 Stage 1 Stage 2 Stage3 Stage 4	2 (0.6) 24 (7.1) 93 (27.4) 24 (7.1) 168 (49.6)
**SES**	Low Mid High	121 (35.7) 126 (37.2) 92 (27.1)
**Diagnosis**	Yes No	162 (47.8) 177 (52.2)
**Chemotherapy**	Yes No	176 (51.9) 163 (48.1)
**Surgery**	Yes No	67 (19.8) 272 (80.2)
**Radiation therapy**	Yes No	91 (26.8) 248 (73.2)
**Palliative care**	Yes No	64 (18.9) 275 (81.1)
**Status**	Alive (based on latest visit at Capital Heath) Dead	25 (7.4) 314 (92.6)
**Type Recurrence**	Residual Local Metastatic disease None (disease free)	274 (80.8) 3 (0.9) 22 (6.5) 30 (8.8)
**Average age of subject (years)**	Mean ± st. dev.	70.9 ± 11.9
**Overall survival (months)**	Mean ± st. dev.	11.0 ± 21.2

**Table 3:** Overall survival by SES or race. Overall survival (OS, in months, mean ± st. dev.) grouped by SES or race. The data demonstrate a significantly lower OS in subjects who live in a low- or mid-SES area compared to individuals who live in an area classified as having a high SES. Similarly, Whites have a significantly longer OS compared to other racial groups. Analysis of variance (ANOVA) was performed to detect significant differences.

SES (ANOVA, *p =* 0.013)	Low	8.7 ± 15.1
	Mid	9.2 ± 14.2
	High	16.6 ± 32.5
Race (ANOVA, *p =* 0.042)	White	13.4 ± 25.5
	African American	7.5 ± 11.7
	Asian	5.8 ± 2.4

**Table 4:** **Utilization of services by SES or race.** Distribution of the number of subjects, grouped by SES or race, who received one or more medical or surgical services. Comparative statistics (chi-square) were performed on the total sample, including subjects who did not receive any type of these services. A significantly different and non-random distribution (*) was detected (chi-square) between SES and receiving or not receiving radiation therapy. A significantly different and non-random distribution (*) was also detected in subjects based on race and undergoing or not undergoing surgical treatment.

Type of Service	Number of Subjects in Each SES Group Who Received (Yes) or Did Not Receive (No) a Medical or Surgical Service	*p*-Value (Chi-Square)
Diagnosis	Low, Yes n = 63/No n = 58 Mid, n = 57/No n = 69 High, n = 42/No n = 50	0.50
Chemotherapy	Low, Yes n = 62, No, n = 59 Mid, Yes, n = 64/No, n = 62 High, Yes, n = 50/No, n = 42	0.86
Surgery	Low, Yes, n = 18/No, n = 103 Mid, Yes, n = 26/No, n = 100 High Yes, n = 23/No, n = 69	0.18
Radiation therapy	Low, Yes, n = 29/No, n = 92 Mid, Yes, n = 28/No, n = 98 High, Yes, n = 34/No, n = 58	0.04 *
Palliative care	Low, Yes, n = 28/No, n = 93 Mid, Yes, n = 24/No, n = 102 High, Yes, n = 12/No, n = 80	0.18
	**Number of Subjects in Each Racial Group Who Received (Yes) or Did Not Receive (No) a Medical or Surgical Service**	
Diagnosis	White, Yes, n = 91/No, n = 113 African American, Yes, n = 67/No, n = 63 Asian, Yes, n = 4/No, n = 1	0.16
Chemotherapy	White, Yes, n = 113/No, n = 91 African American, Yes, n = 60/No, n = 70 Asian, Yes, n = 3/No, n = 2	0.24
Surgery	White, Yes, n = 50/No, n = 154 African American, Yes, n = 17/No, n = 113 Asian, Yes, n = 0/No, n = 5	0.02*
Radiation therapy	White, Yes, n = 62/No, n = 142 African American, Yes, n = 28/No, n = 102 Asian, Yes, n = 1/No, n = 4	0.19
Palliative care	White, Yes, n = 33/No, n = 171 African American, Yes, n = 30/No, n = 106 Asian, Yes, n = 1/No, n = 4	0.29

**Table 5:** (**A**) Insurance and SES. Distribution of the subjects based on the type of insurance and SES. A comparative statistic for nominal data (counts) was used to detect significant differences between group (chi-square and *p =* 0.007). The data indicate that there is a significant difference between SES groups and type of insurance. (**B**) Insurance and race. Distribution of the subjects based on the type of insurance and race. A comparative statistic for nominal data (counts) was used to detect significant differences between group (chi-square *p =* 0.001). The data indicate that there is a significant difference between race and type of insurance.

**(A)**			
**Type of Insurance**	**Low**	**Mid**	**High**
Insured, but not specified	13 (11%)	10 (8%)	10 (11%)
Medicare	53 (44%)	80 (64%)	55 (60%)
Medicaid	9 (7%)	9 (7%)	0 (0%)
Private	29 (24%)	20 (16%)	22 (24%)
Not insured	17 (14%)	7 (6%)	5 (5%)
Total	121 (100%)	126 (100%)	92 (100%)
**(B)**			
**Type of Insurance**	**White**	**Black**	**Asian**
Insured but not specified	16 (8%)	17 (13%)	0 (0%)
Medicare	127 (62%)	57 (44%)	4 (80%)
Medicaid	4 (2%)	14 (11%)	0 (0%)
Private	45 (22%)	26 (20%)	0 (0%)
Not insured	12 (6%)	16 (12%)	1 (20%)
Total	204 (100%)	130 (100%)	5 (100%)

**Table 6:** (**A**)**.** Cancer stage and SES. The distribution of the number of subjects and tumor stage at the time of diagnosis. A comparative statistic for nominal data (counts) was used to detect significant differences. No significant differences were noted (chi-square, *p* = 0.53), indicating that stage at the time of diagnosis is not differentially distributed according to SES. Percentages are omitted for clarity. (**B**). Cancer stage and race. The distribution of the number of subjects by race and tumor stage at the time of diagnosis. A comparative statistic for nominal data (counts) was used to detect significant differences. No significant differences were noted (chi-square, *p =* 0.31), indicating that stage at the time of diagnosis is not differentially distributed when race was considered. Percentages are omitted for clarity.

(**A**)									
			Stage						Total
			Stage 0	Stage 1	Stage 2	Stage 3	Stage 4	Missing	
SES	Low		1	9	30	6	68	7	121
	Mid		1	7	33	11	59	15	126
	High		0	8	30	7	41	6	92
Total			2	24	93	24	168	28	339
(**B**)									
			Stage						Total
			Stage 0	Stage 1	Stage 2	Stage 3	Stage 4	Missing	
Race		White	2	15	62	18	90	17	204
		African American	0	9	30	5	76	10	130
		Asian	0	0	1	1	2	1	5
Total			2	24	93	24	168	28	339

## Data Availability

We used internal data abstracted from the CHS Cancer Registry.

## Abbreviations

AGI	Adjusted Gross Income
AA	African American
OS	Overall Survival
SES	Socioeconomic Status
US	United States
